# Coat protein is responsible for tomato leaf curl New Delhi virus pathogenicity in tomato

**DOI:** 10.3389/fpls.2023.1206255

**Published:** 2023-07-10

**Authors:** Thuy T. B. Vo, Aamir Lal, Bupi Nattanong, Marjia Tabassum, Muhammad Amir Qureshi, Elisa Troiano, Giuseppe Parrella, Eui-Joon Kil, Sukchan Lee

**Affiliations:** ^1^Department of Integrative Biotechnology, Sungkyunkwan University, Suwon, Republic of Korea; ^2^Agriculture Science and Technology Research Institute, Andong National University, Andong, Republic of Korea; ^3^Department of Biology, Agriculture and Food Sciences, Institute for Sustainable Plant Protection of the National Research Council (IPSP-CNR), Portici, Italy; ^4^Department of Plant Medicals, Andong National University, Andong, Republic of Korea

**Keywords:** coat protein, ToLCNDV strain, host interaction, mutant infectious clones, infectivity assay

## Abstract

*Tomato leaf curl New Delhi virus* (ToLCNDV), a bipartite *Begomovirus* belonging to the family *Geminiviridae*, causes severe damage to many economically important crops worldwide. In the present study, pathogenicity of Asian (ToLCNDV-In from Pakistan) and Mediterranean isolates (ToLCNDV-ES from Italy) were examined using infectious clones in tomato plants. Only ToLCNDV-In could infect the three tomato cultivars, whereas ToLCNDV-ES could not. Genome-exchange of the two ToLCNDVs revealed the ToLCNDV DNA-A segment as the main factor for ToLCNDV infectivity in tomato. In addition, serial clones with chimeric ToLCNDV-In A and ToLCNDV-ES A genome segments were generated to identify the region determining viral infectivity in tomatoes. A chimeric clone carrying the ToLCNDV-In coat protein (CP) exhibited pathogenic adaptation in tomatoes, indicating that the CP of ToLCNDV is essential for its infectivity. Analyses of infectious clones carrying a single amino acid substitution revealed that amino acid at position 143 of the CP is critical for ToLCNDV infectivity in tomatoes. To better understand the molecular basis whereby CP function in pathogenicity, a yeast two-hybrid screen of a tomato cDNA library was performed using CPs as bait. The hybrid results showed different interactions between the two CPs and Ring finger protein 44-like in the tomato genome. The relative expression levels of upstream and downstream genes and Ring finger 44-like genes were measured using quantitative reverse transcription PCR (RT-qPCR) and compared to those of control plants. This is the first study to compare the biological features of the two ToLCNDV strains related to viral pathogenicity in the same host plant. Our results provide a foundation for elucidating the molecular mechanisms underlying ToLCNDV infection in tomatoes.

## 1 Introduction

Tomatoes (*Solanum lycopersicum* L.) are one of the most important crops in the world, with a high economic value owing to their composition (Hanssen et al., 2010). Many diseases affect tomato production, and approximately half are caused by plant viruses, with at least 312 viruses, satellite viruses, or viroid species associated with tomatoes (Rivarez et al., 2021). Begomoviruses belonging to the *Geminiviridae* family have been identified as prevalent limiting factors for tomato cultivation in many regions (Mabvakure et al., 2016), and tomato leaf curl New Delhi virus (ToLCNDV) is one of the most destructive begomoviruses.

ToLCNDV has a bipartite genome structure (Moriones et al., 2017). In 1995, ToLCNDV was first reported in tomatoes in India (Padidam et al., 1995a) and then spread to cucurbits in the Mediterranean Basin in 2012 (Juárez et al., 2014; Mnari-Hattab et al., 2015; Parrella et al., 2018; Orfanidou et al., 2019). In addition to its wide host range (Srivastava et al., 2016; Jamil et al., 2017; Venkataravanappa et al., 2018; Sharma et al., 2021), this virus also possesses other dangerous characteristics, such as multi-mode transmissibility, including mechanical and seed transmission (López et al., 2015; Kil et al., 2020). Thus, ToLCNDV has emerged as a serious threat to many important crops because of its rapid spread and the extent of the outbreak, even in countries with different geographical locations, thanks mainly to its efficient vector, the *Bemisia tabaci* (Bertin et al., 2018; Bertin et al., 2021). Previous research reported that two strains of ToLCNDV from Indian subcontinent and the Mediterranean Basin presented distinct pathogenicity in tomatoes (Brown et al., 2015; Fortes et al., 2016). Tomato plants infected with the ToLCNDV Indian strain (ToLCNDV-In) exhibited severe symptoms, such as stunted growth, leaflets curled upward and downward, slight chlorosis and yellowing, crumpling, and mosaic/mottling (Singh et al., 2015). Regardless, the ToLCNDV Mediterranean strain (ToLCNDV-ES strain) has primarily adapted to cucurbits, while evolutionary dynamics of this strain observed in Italy, have apparently led to the selection of two subgroups: subgroup I, which is not able to infect the tomato (Vo et al., 2022b; Troiano and Parrella, 2023) and subgroup II which instead infecting tomatoes, although with great difficulty, as evidenced by a low incidence and light symptoms in the field in this plant (Fortes et al., 2016; Ruiz et al., 2017; Yamamoto et al., 2021). Overall, these characteristics indicate that tomato is not a permissive host for ToLCNDV-ES, whereas ToLCNDV isolates from Asia are regarded as a threat to this plant species. However, the causes for these characteristics remain largely unknown. However, to date, there have been no studies comparing the biological characteristics and the molecular mechanisms of different strains of ToLCNDV in the same host species.

In this study, two ToLCNDV isolates, belonging to Asian and Mediterranean strains respectively, were confirmed to have differential infectivity in tomatoes using infectious clones that were successfully constructed previously (Vo et al., 2022a). ToLCNDV isolates from Pakistan, designated as ToLCNDV-In, induced severe symptoms, unlike ToLCNDV-ES isolates from Italy, which are not adapted to tomatoes (Vo et al., 2022b; Troiano and Parrella, 2023). To identify the pathogenicity determinants of ToLCNDV in tomatoes, genomic segments were exchanged between the two isolates and the hybrids were inoculated into different tomato cultivars. After identifying DNA-A as the main factor for ToLCNDV infectivity in tomato, we generated numerous mutant clones by altering six ORFs and the intergenic region (IR) between the two strains. An infectivity assay showed that the clone carrying coat protein (CP) was responsible for ToLCNDV pathogenicity in tomatoes. Site-directed mutagenesis of CP was performed to examine the role of amino acid residues in ToLCNDV infection. The amino acid at position 143 of the CP was found to play a critical role in tomato ToLCNDV-ES infectivity. Yeast two-hybrid screening was performed to examine the interaction between the CP of each ToLCNDV isolate and tomato host proteins. The hybrid assay results showed different interactions between the two ToLCNDV CPs with Ring-finger protein 44-like. This interaction may explain the difference in pathogenicity of the two ToLCNDV strains in tomatoes. This is the first in-depth study that compares biological characteristics of two ToLCNDV strains in the same host plant and identifies the key viral infectivity determinants.

## 2 Materials and methods

### 2.1 Virus sources and plant growth condition

A ToLCNDV isolated from Pakistan, belonging to ToLCNDV-India strain group (here designated as ToLCNDV-In) and the Italian isolate 45/16, belonging to subgroup I of ToLCNDV-ES strain group (here designated as ToLCNDV-ES), were used in the present study (Parrella et al., 2018; Vo et al., 2022a).

Infectious clones were successfully generated in a previous study (Vo et al., 2022a). *Nicotiana benthamiana* and three different tomato cultivars including ‘San Pedro’ from Italy, ‘Seogwang’ from Korea, and ‘Moneymaker’ were cultivated in a growth chamber with 16 h light/8 h dark cycles at 22–28 °C

### 2.2 Construction of chimeric and site-directed mutant clones of two ToLCNDV isolates

Subgenomic components were swapped to form two combinations, ToLCNDV-In DNA-A + ToLCNDV-ES DNA-B and ToLCNDV-ES DNA-A + ToLCNDV-In DNA-B (hereafter designate as ToLCNDV-In-A, ToLCNDV-In-B, ToLCNDV-ES-A and ToLCNDV-ES-B respectively) using an approach similar to other agroinoculations (Vo et al., 2022a). Seven chimeric sequences corresponding to six ORF and the IR of DNA-A components were synthesized by swapping the ToLCNDV-ES sequence on the ToLCNDV-In backbone (Macrogen, Korea) based on the wild-type sequence of the two ToLCNDV isolates with tandem repeat constructs (**Figure S1**). Plasmids containing swapped sequences were digested with *Hin*dIII/*Spe*I and ligated into the pCAMBIA 1303 vector to generate recombinant plasmids. After transformation of *Agrobacterium tumefaciens* strain GV3101 using the freeze-thaw transformation method (Weigel and Glazebrook, 2006), the infectivity of all the chimeric clones was tested on *N.benthamiana* and tomato plants.

Site-directed mutants of CP were produced using Q5^®^ Site-Directed Mutagenesis Kit (New England Biolabs, Ipswich, MA, USA) following the manufacturer’s instructions. Based on the different amino acids in the V1 sequence between the two isolates using multiple alignments, 16-point mutant clones were generated using the designed primer sets (**Table S1**) with extracted viral DNA from infected leaves as a template. All mutant sequences were confirmed by sequencing, which were then transformed into *Agrobacterium* to generate infectious clones using the same methodology.

### 2.3 Virus inoculation

*A.tumefaciens* harboring each viral clone was grown in 20 mL of LB broth medium containing kanamycin (50 µg/mL), rifampicin (50 µg/mL), and gentamycin (50 µg/mL) for 24 h at 28°C with shaking until the optical density (OD) reached 1.0 at 600 nm. Activated cells were pelleted by centrifuging and resuspended in infiltration buffer (10 mM MgCl_2_, 10 mM MES, 200 µM acetosyringone). *A. tumefaciens* strains containing the recombinant plasmids ToLCNDV-In-A/ToLCNDV-In-B, ToLCNDV-ES-A/ToLCNDV-ES-B, ToLCNDV-In-A/ToLCNDV-ES-B, and ToLCNDV-In-B/ToLCNDV-ES-A were mixed in a 1:1 ratio, and the strains containing chimeric and site-directed mutant clones were mixed in equal proportions. Cell suspensions were used for the infectivity assay of three-week-old tomato plants by pinpricking the main apical shoot.

### 2.4 Detection of two ToLCNDV isolates accumulation

Inoculated leaf samples were collected 21 days post-inoculation (dpi) to confirm the viral infection. Genomic DNA was extracted and used for PCR amplification with specific primer sets, as described previously (Vo et al., 2022a). To confirm viral replication, Southern blotting was performed as previously described (Southern, 2006). Briefly, after electrophoresis, DNA was transferred to nylon membranes and hybridized with a probe containing [α^32^P]-dCTP at 65°C for 16 h. The membrane was washed and exposed to an X-ray film.

Quantitative PCR (qPCR) was performed on the agroinoculated plants at 21 dpi to determine the viral titer. Total DNA from different tissues, including new leaves, stems, and roots of the three plants, was extracted and used as a template for qPCR following our previous study (Vo et al., 2022b).

### 2.5 Yeast two hybrid assay

To identify the interactions between the CPs of each isolate and the host factors, yeast two-hybrid screening of the tomato cDNA library was conducted. For cDNA library construction, total RNA from tomato leaves was isolated using the RNeasy Plant Mini Kit (Qiagen, Hilden, Germany) according to the manufacturer’s instructions. The integrity of the total RNA was analyzed using 1% agarose gel electrophoresis and the concentration was determined using a BioTek Epoch Microplate spectrophotometer (BioTek, Winoosku, VT, USA). Then, this RNA was used to construct the Y2H tomato cDNA library by Panbionet (Pohang, Korea). The CP (V1) encoding gene of each isolate was introduced into the pGBKT7 (referred to as In-V1 and ES-V1) vector, which contained a GAL4 DNA-binding domain, kanamycin resistance for selection in *Escherichia coli*, and a TRP1 nutritional marker for selection in yeast. Two plasmids, pGBKT7-In-V1 and pGBKT7-ES-V1, were generated as baits in the GAL4-based two-hybrid system for CPs of ToLCNDV-ES and ToLCNDV-In isolates, respectively. Bait vectors were checked for toxicity and autoactivation in yeast before processing the hybrids. The bait was then hybridized with a cDNA library to identify CP-interaction proteins in tomatoes by Panbionet.

To confirm the interaction between each ToLCNDV CP and the host protein, yeast two-hybrid assays were conducted using a modified lithium acetate yeast transformation (Thompson et al., 1998). Interaction of different host genes including Cathepsin B-like protease 3 (XM_004233173), NAD(P)H-quinone oxidoreductase subunit M (XM_004237313), Ribulose bisphosphate carboxylase small chain 3B (NM_001309210), protein phosphatase 2C (NM_001247571), LOW PSII ACCUMULATION (XM_004247389), NAD(P)H-quinone oxidoreductase subunit M (XM_004237313), and RING finger protein 44- like (XM_004243217) was examined with In-V1 and ES-V1. Prey, including host genes, were cloned into the pGADT7 vector and co-transformed with bait vector pGBKT7 carrying different CPs into *Saccharomyces cerevisiae* strain Y2H Gold. The transformed yeast cells were plated on three nutrient dropout media: SD/-Trp/-Leu (DDO), SD/-Trp/-Leu/-His (TDO), and SD/-Trp/-Leu/-Ade/-His (QDO). The plasmids pGBKT7-53 (Gal4 DNA-BD fused with murine 53) and pGBKT7-Lam (Gal4 BD fused with lamin) were co-transformed with pGADT7-T encoding the Gal4 AD fused with the SV40 large T-antigen and used as positive and negative controls, respectively.

### 2.6 Relative expression of candidate genes by RT-qPCR

For RT-qPCR, cDNA was synthesized from 1µg of total RNA using Oligo dT primers and Moloney murine leukemia virus (MMLV) reverse transcriptase (Bioneer, Daejeon, Korea). The reaction was performed using TB Green® Premix Ex Taq™ II (Tli RNaseH Plus; TaKaRa Bio, Shiga, Japan) with the primer sets (**Table S2**). Forty cycles of PCR were performed using a Rotor-Gene Q thermocycler (Qiagen) under the following conditions: 10 s denaturation at 95°C, 15 s annealing at 60°C, and 20 s polymerization at 72°C. Elongation 1α (EF1α) gene was used for internal normalization and each reaction was replicated three times. Data analyses were conducted using the 2^−ΔΔCt^ method (Livak and Schmittgen, 2001). Statistical analyses were performed using the t-test in the GraphPad Prism software (GraphPad Software, Boston, MA, USA).

## 3 Results

### 3.1 Determination of the virulence region using two ToLCNDV infectious clones

**Figure 1:**
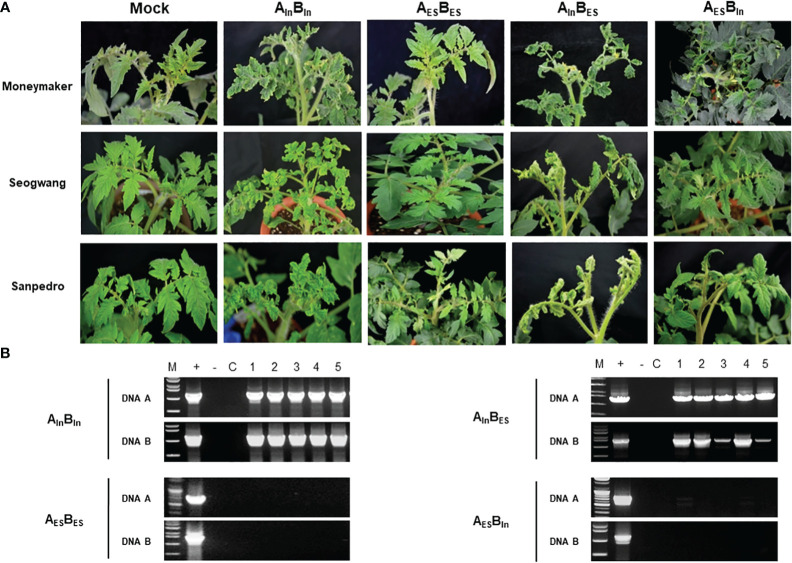
Tomato plants infected with swapped ToLCNDV constructs. **(A)** Disease phenotype induced by two ToLCNDV constructs including the original combination (ToLCNDV-ES DNA-A (A_ES_) + DNA B (B_ES_) and ToLCNDV-In DNA-A (A_In_) + DNA B (B_In_) and swapped subgenome combination (A_In_B_ES_ or A_ES_B_In_)at 21 dpi. **(B)** PCR results of viral DNA in systemically infected tomato leaves. Lane M: maker, lane +: positive control, lane −: negative control, lane C: mock plant, lane 1–5: inoculated plants.

Infectivity assays revealed that the ToLCNDV-In infectious clone could infect tomatoes, whereas the ToLCNDV-ES clone was not adapted to this crop (**Figure 1**). Typical symptoms, including leaf curl, mosaic, and stunting, were induced in ToLCNDV-In-infected tomatoes, whereas a normal phenotype was observed in ToLCNDV-ES-inoculated plants for all the three tomato cultivars (Moneymaker, Seogwang, and San Pedro) (**Figure 1A**). The PCR detection results were consistent with the appearance of symptoms in the inoculated tomatoes (**Figure 1B**).

To determine the genomic region responsible for ToLCNDV pathogenicity in tomato, the subgenomes of the two isolates were swapped by mixing DNA components as follows: ToLCNDV-ES-A + ToLCNDV-In-B (A_ES_B_In_) and ToLCNDV-In-A + ToLCNDV-ES-B (A_In_B_ES_) and processed, and original ToLCNDV-ES-A/B (A_ES_B_ES_) and ToLCNDV-In-A/B (A_In_B_In_) were used as positive and negative control, respectively. The results revealed that only ToLCNDV-In-A supplemented with either ToLCNDV-In-B or ToLCNDV-ES-B could infect tomatoes (**Table 1**). Infected plants exhibited a typical disease phenotype at 21 dpi (**Figure 1A**). Furthermore, the viral genome was detected in all symptomatic plants inoculated with A_In_B_In_ and A_In_B_ES_ using PCR amplification with specific primers. No infection was observed with A_ES_B_ES_ or A_ES_B_In_ (**Figure 1B**). Virulence was only observed when tomatoes were co-inoculated with ToLCNDV-In-A, demonstrating that the DNA-A component of ToLCNDV-In was responsible for infectivity in tomatoes.

**Table 1:** Infectivity of experiment ToLCNDV clones in *Nicotiana benthamiana* and tomato.

Experiment	IC	*N. benthamiana*		Tomato	
		Infectivity*	Symptoms	Infectivity*	Symptoms
Subgenome swapped	Mock	0/6	–	0/9	–
	A_ES_B_ES_	6/6	Leaf curl, mosaic, stunting	0/9	–
	A_In_B_In_	6/6	Leaf curl, mosaic, stunting	9/9	Leaf curl, yellow mosaic, pickering
	A_ES_B_In_	6/6	Leaf curl, mosaic, stunting	0/9	–
	A_In_B_ES_	6/6	Leaf curl, mosaic, stunting	9/9	Leaf curl, yellow mosaic, pickering
Chimeric	In_IR_ES	6/6	Leaf curl, mosaic, stunting	0/8	–
	In_C1_ES	6/6	Leaf curl, mosaic, stunting	0/8	–
	In_C2_ES	6/6	Leaf curl, mosaic, stunting	0/8	–
	In_C3_ES	6/6	Leaf curl, mosaic, stunting	0/8	–
	In_C4_ES	6/6	Leaf curl, mosaic, stunting	0/8	–
	In_V1_ES	6/6	Leaf curl, mosaic, stunting	6/8	Moderate leaf curling
	In_V2_ES	6/6	Leaf curl, mosaic, stunting	0/8	–
	ES_V1_In	6/6	Leaf curl, mosaic, stunting	0/8	–

*Infected plants/inoculated plants.

### 3.2 Coat protein is responsible for ToLCNDV infectivity in tomato

**Figure 2:**
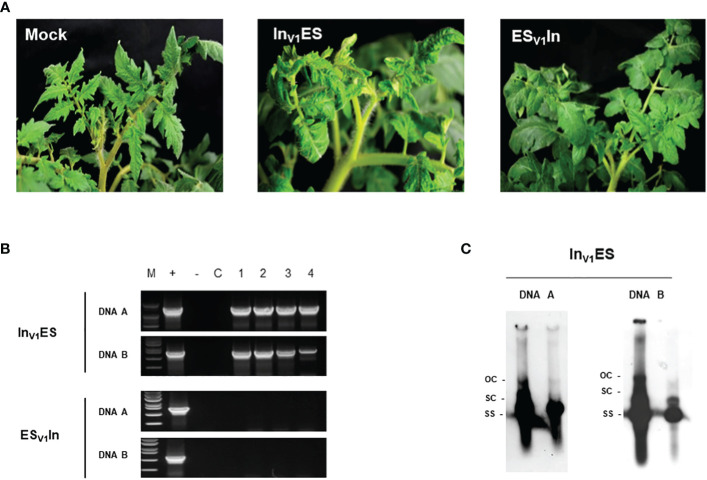
Infectivity of AV1 chimeric constructs in tomato. **(A)** Visual symptom of two AV1 constructs (In_V1_ES: ToLCNDV-In CP in ToLCNDV-ES backbone; ES_V1_In: ToLCNDV-ES CP in ToLCNDV-In backbone) at 21 dpi. **(B)** PCR detection of viral DNA in infected tomatoes. Lane M: marker, Lane +: positive control, lane −: negative control, lane C: mock plant, lane 1–4: inoculated plants. **(C)** Southern blot hybridization results of In_V1_ES chimera clone; the amplicon from PCR detection was used as probe.

To identify the gene(s) that determine tomato infection by ToLCNDV-In, we synthesized seven chimeric constructs by substituting genomic regions containing C1 (Rep), C2 (TrAP), C3 (REn), C4, V1 (CP), V2, and IR of ToLCNDV-In into the ToLCNDV-ES backbone (abbreviated as In_IR_ES, In_C1_ES, In_C2_ES, In_C3_ES, In_C4_ES, In_V1_ES, and In_V2_ES). After three weeks of inoculation, chimeric construct In_V1_ES induced moderate leaf curl symptoms, consistent with the PCR amplification results, whereas visible symptoms and viral DNA were not detected in all other constructs in tomatoes (**Figure 2A**; **Table 1**). A chimeric clone harboring ToLCNDV-ES V1 in the ToLCNDV-In backbone (ES_V1_In) was constructed to confirm the role of CP in ToLCNDV infectivity. There was no virus accumulation in symptomless ES_V1_In-inoculated plants, as confirmed by PCR (**Figure 2B**). According to the Southern hybridization results, open circular and super-coiled forms (double–stranded DNA), as well as a single-stranded form of both DNA components indicated that the chimeric In_V1_ES clone can replicate in tomato (**Figure 2C**). Taken together, these results suggest that the CP is responsible for the infectivity in tomatoes of the ToLCNDV-In and of its chimeric construct In_V1_ES.

### 3.3 Effect of a single amino acid mutation on coat protein on virus infectivity

**Figure 3:**
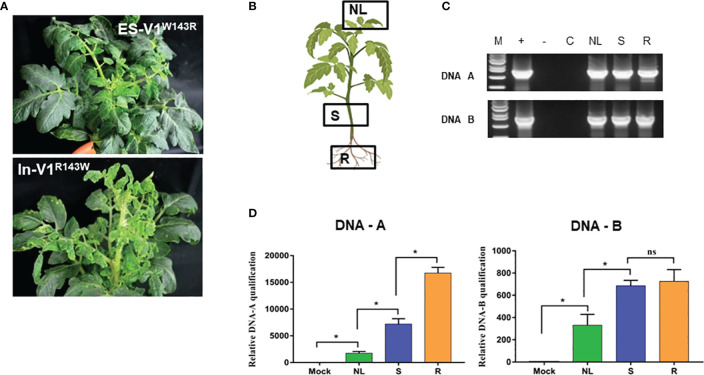
Infectivity of amino acid 143 position point mutant clones of coat protein of ToLCNDV. **(A)** Phenotype of point mutant constructs (ES-V1^W143R^: the residue tryptophan at amino acid 143 was changed to arginine in ToLCNDV-ES CP and In-V1^R143W^: arginine was changed to tryptophan in ToLCNDV-In CP) in tomato. **(B, C)** Sampling position and PCR results in different tissues, Lane M: positive control, Lane −: negative control, Lane C: mock plant, NL, new leaf; S, stem; R, root. **(D)** Relative viral titer in different tissues. The bar graph indicate the mean ± standard deviation (n = 3). The statistical comparison was performed with the unpaired t-test: *p <0.05, ns, not significant.

To determine the role of different amino acids in the CPs in pathogenicity in tomato, eight site-directed mutant constructs were generated. These included ES-V1^S2A^ (serine residue at amino acid 2 was changed to alanine), ES-V1^T29A^ (threonine residue at amino acid 29 was changed to alanine), ES-V1^S32V^ (serine residue was changed to valine at amino acid 32), ES-V1^R42K^ (arginine residue was changed to lysine at amino acid 42), ES-V1^W143R^ (residue tryptophan at amino acid 143 was changed to arginine), ES-V1^T148S^, (serine replaced threonine at amino acid 148), ES-V1^S193C^ (cysteine residue replaced serine at amino acid 193), ES-V1^R194K^ (arginine residue was changed to lysine at amino acid 194) based on alignment of V1 full length of ToLCNDV-ES. At contrary, mutants In-V1^A2S^, In-V1^A29T^, In-V1^V32S^, In-V1^K42R^, In-V1^R143W^, In-V1^S148T^, In-V1^C193S^, and In-V1^K194R^were generated based on ToLCNDV-In V1 sequence and their infectious clones were constructed (**Figure S2**). All mutant infectious clones were agroinoculated into tomato plants to confirm their infectivity with the wild-type ToLCNDV clones. A symptomless phenotype was observed in all plants inoculated with either mutant or wild-type ToLCNDV-ES clone after three weeks of inoculation (**Figure 3A**; **Table S3**). Nevertheless, viral DNA was only detected in ES-V1^W143R^-inoculated plants. Three different tissues, upper leaves, stems, and roots of asymptomatic infected plants, were examined for viral presence to determine the movement of the ToLCNDV mutant clone (**Figure 3B**). PCR results showed that this mutant clone was present in all tested tissues, indicating that ToLCNDV-ES with a single mutation at amino acid 143 position of the CP could replicate and spread systemic infection to other tissues (**Figure 3C**). In addition, qPCR was performed to quantify the relative viral accumulation of the mutant clone in various tomato tissues after infection. No viral DNA accumulated in the mock-treated plants, whereas there was a high level of mutant ES-V1^W143R^ in all infected tissues, especially in the roots (**Figure 3D**). In addition, all mutant clones of different amino acids, including amino acid 143 on the ToLCNDV-In CP, showed infectivity and induced typical symptoms in tomatoes. The detectable PCR results (data not shown) and symptomatic phenotype of inoculated plants indicated that the mutation at the amino acid 143 position did not have a major effect on ToLCNDV-In pathogenicity but was essential for ToLCNDV-ES infectivity in tomato, even when no disease symptoms were induced.

In previous reports, some ToLCNDVs isolated from Spain also showed weak adaptation to asymptomatic tomatoes, even though a few plants showed slight vein yellowing only in the lower leaves (Fortes et al., 2016; Yamamoto et al., 2021). Comparison of amino acid sequences among CPs of Spanish ToLCNDV and the two isolates in this study revealed that both isolates from Mediterranean only differ in amino acid 143, suggesting that this amino acid plays an important role in ToLCNDV-ES’s ability to infect tomatoes.

### 3.4 Interaction of ToLCNDV coat protein and host factors

Yeast two-hybrid screening was conducted to determine the interaction between the CP of each ToLCNDV isolate and host proteins. In the screening, only 26 of 53 and 10 of 25 candidates truly interacted with ToLCNDV-In CP and ToLCNDV-ES CP, respectively. After sequencing, five host proteins including Cathepsin B-like protease 3, NAD(P)H-quinone oxidoreductase subunit M,Ribulose bisphosphate carboxylase small chain 3B (RBCS4), Protein phosphatase 2C (DIG3), and Protein LOW PSII ACCUMULATION 1 interacted with ToLCNDV-In CP, whereas CP of ToLCNDV-ES interacted with NAD(P)H-quinone oxidoreductase subunit M protein, RING finger protein 44- like, GAGA- binding transcriptional activator, protein CROWDED NUCLEI 4, 1-D-deoxyxylulose 5-phosphate synthase, and thaumatin-like protein 1b (**Table 2**). Both baits interacted with the same NAD(P)H-quinone oxidoreductase subunit M protein, a flavoenzyme that serves as a quinone reductase, in connection with the conjugation reactions of hydroquinones involved in detoxification pathways (Sellés Vidal et al., 2018).

**Table 2:** Host proteins interaction with CP of each ToLCNDV isolates, as determined by Y2H screening.

Isolates	Hit *	Host genes	NCBI accession No.
In- CP	14	Cathepsin B-like protease 3	XM_004233173
	1	NAD(P)H-quinone oxidoreductase subunit M	XM_004237313
	1	Ribulose bisphosphate carboxylase small chain 3B, chloroplastic-like (RBCS4)	NM_001309210
	1	Protein phosphatase 2C (DIG3)	NM_001247571
	1	Protein LOW PSII ACCUMULATION 1	XM_004247389
ES-CP	1	NAD(P)H-quinone oxidoreductase subunit M	XM_004237313
	1	RING finger protein 44- like	XM_004243217
	1	GAGA-binding transcriptional activator (BBR/BPC2)	NM_001279314
	1	Protein CROWDED NUCLEI 4 (LOC101246104)	XM_004231905
	1	1-D-deoxyxylulose 5-phosphate synthase (DXS)	NM_001247743
	1	Thaumatin-like protein 1b	XM_004229555

*Number of prey identified independently.

**Figure 4:**
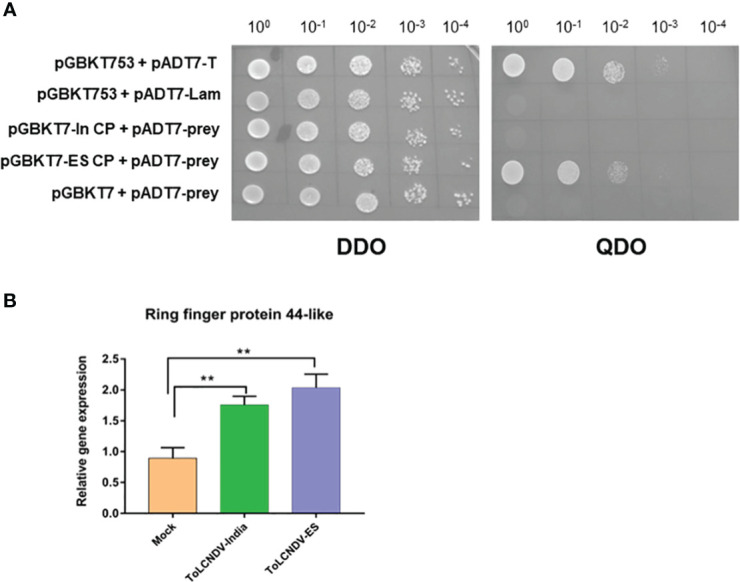
Confirmation of Y2H for Ring finger protein 44-like and its expression by RT-qPCR **(A)** Y2H results when each CP is hybridized with Ring finger protein 44-like. CP of each ToLCNDV as bait and Ring finger protein 44-like as prey; pGBKT53 x pADT7-T is positive control, pGBKT53x pADT7-Lam is negative control. Two selection media are DDO (double dropout SD/-Trp/-Leu) and QDO (Quadruple dropout SD/-Trp/-Leu/-His/-Ade). **(B)** Relative expression change of Ring finger protein 44-like in different inoculated leaves at 21 dpi. The statistical comparison was performed with the unpaired t-test: **p <0.01.

To verify the interaction between the two CPs and host genes derived from the screening list, baits containing ToLCNDV-In CP and ToLCNDV-ES CP were co-transfected with the prey vector harboring the host genes in the S. *cerevisiae* strain Y2H Gold. Transfected yeasts were grown under different nutrient deficiencies (DDO, TDO, and QDO) with serial dilutions to confirm the interactions. After three days of incubation, the results showed that all captured host proteins interacted with ToLCNDV-In CP and ToLCNDV-ES, except for Ring finger protein 44-like. Interestingly, Ring-finger protein 44- like interacted only with ToLCNDV-ES CP and did not show any binding with ToLCNDV-In CP bait (**Figure 4A**). The yeast co-transfected with ES CP bait and ring finger prey grew on three nutrient-deficient nutrient media, while yeast co-transfected with ToLCNDV-In CP did not produce any colonies on DDO and QDO media, indicating that there was no interaction between ToLCNDV-In CP and this host protein.

**Figure 5:**
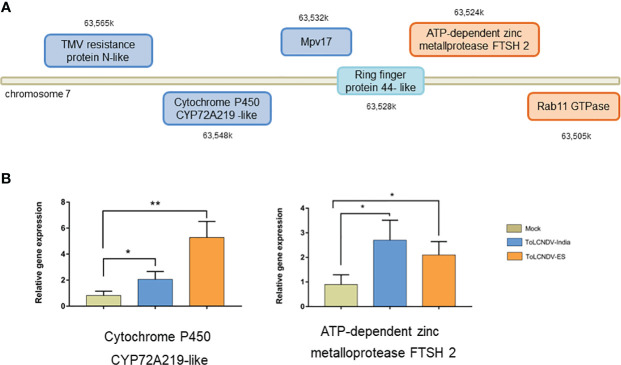
Relative expression of upstream and downstream genes for Ring-finger protein 44-like, as determined by RT-qPCR. **(A)** Position of chosen genes in upstream (blue rectangle) and downstream (orange rectangle) region. Their relative expression was checked in plants inoculated with different isolates including mock, ToLCNDV-In, and ToLCNDV-ES; however only one upstream gene and one downstream gene showed significant difference **(B)**. The statistical comparison was performed with the unpaired t-test: *p<0.05, **p <0.01.

We compared the Ring-finger protein 44-like transcript levels in tomato leaves inoculated with ToLCNDV-In, ToLCNDV-ES and *A. tumefaciens* GV3101 (mock) using RT-qPCR. The relative expression of this protein in ToLCNDV-inoculated leaves was significantly higher than that in GV3101-infiltrated leaves at 21 dpi as shown in **Figure 4B**, suggesting that the expression of Ring-finger protein 44-like was induced by virus inoculation. However, no significant difference in Ring protein expression between the two ToLCNDV clone-inoculated plants suggests that the infectivity of ToLCNDV in tomato is not related only to the accumulation of Ring-finger protein 44-like. We also assessed the expression of five genes in the upstream and downstream regions under these two conditions (**Figure 5**; **Table S4**). RT-qPCR showed increase in expression of cytochrome P450 CYP72A219-like in the upstream region and ATP-dependent zinc metalloprotease FTSH2 in the downstream region with viral infection compared to their expression in mock plants. Expression of upstream genes increased the most in the ToLCNDV-ES-inoculated group. This gene is involved in growth and defense mechanisms by inducing the biosynthesis of defense compounds such as jasmonic acid and flavonoids. These factors also potentially affect ToLCNDV pathogenicity in tomatoes.

## 4 Discussion

Tomato leaf curl New Delhi virus is a devastating pathogen that causes economic losses to many important crops worldwide (Padidam et al., 1995a; Zaidi et al., 2017). This pathogen shows genetic variability and two main strains, including those from Asia and the Mediterranean region, have been described. In the Indian subcontinent, ToLCNDV has been reported to cause severe symptoms and yield losses, particularly in solanaceous crops (e.g. tomato and aubergine), (Hanssen et al., 2010; Kumar et al., 2015). In the Mediterranean region, however, this virus has caused tremendous damage, mainly to cucurbit crops, while exhibiting low pathogenicity to *Solanaceae* plants. The ToLCNDV outbreak caused losses of over 20% of zucchini and melons in Spain and 80% of pumpkins in Italy (Sáez et al., 2017; Panno et al., 2019; Crespo et al., 2020), whereas no significant losses of tomatoes have been reported in these regions due to this virus to date. Most studies have focused on different adaptations in susceptible or resistant/tolerant hosts to identify candidate genes against this virus using molecular techniques or high-throughput sequencing (Kushwaha et al., 2015; Romero-Masegosa et al., 2021; Sáez et al., 2022). To date, no studies have been conducted to compare the biological characteristics and reveal the precise molecular mechanisms by which different ToLCNDV strains display distinct adaptations in the same hosts. In our study, two isolates representing Asian and Mediterranean strains showed significant differences in infectivity in agroinoculated tomato, a predominant host for begomoviruses. The infectious clone of ToLCNDV-In isolated from Pakistan exhibited high pathogenicity with severe symptoms, whereas the ToLCNDV-ES clone from Italy showed no pathogenicity in tomatoes. To identify the genomic determinants causing differences in tomato adaptation between the two ToLCNDV strains, many constructs of swapped clones were generated and tested in various tomato cultivars. Genetic evidence indicated that DNA-A of ToLCNDV-In plays a critical role in infectivity in tomatoes (**Figure 1**). In addition, the results derived from a series of chimeric viral clones revealed that CP in DNA-A is the key determinant required for the infectivity in tomato. Substituting the CP of ToLCNDV-In within the ToLCNDV-ES backbone resulted in adaptation in tomato, whereas at contrary ToLCNDV-In with the alternative ToLCNDV-ES CP (**Figure 2**) could not infect tomato. These results complement previous reports indicating that the NSP in DNA B is a symptom determinant of ToLCNDV isolated from Pakistan (Hussain et al., 2005). Both CP and NSP perform their role at different stages of viral invasion. CP is a multifunctional protein that serves to encapsidate the geminate particle, allows the transmission by *B. tabaci*, target the nucleus and export the nuclear viral genome (Harrison et al., 2002). Also, CP is known to be essential for the systemic invasion of monopartite begomoviruses, such as TYLCV, but is not essential for bipartite begomoviruses (Gardiner et al., 1988; Padidam et al., 1995b), indicating that CP plays a more important role in propagation than cell-to-cell movement of viruses inside plants. NSP plays a key role in cell-to-cell movement to spread virus to other tissues in many bipartite begomoviruses (Gafni and Epel, 2002). It is possible that when ToLCNDV-ES CP was restricted in the nascent viral production process for unknown reasons, no infection occurred and NSP could not continue to initiate systemic infection. This theory was also supported by recent field and experimental evidence of complementation between an Italian isolate of ToLCNDV-ES (belonging to subgroup I) and TYLCV, thanks to which ToLCNDV-ES became infectious in tomatoes (Vo et al., 2022b).

On the other hand, Indian isolate CP on ES backbone helped in viral propagation and resulted in systemic infection of tomato through movement proteins. However, the connection with ToLCNDV-ES movement proteins is inefficient compared with original ToLCNDV-In DNA B component, which resulted in the moderate disease phenotype (leaf curling) without severe symptom like wild type ToLCNDV-In. Thus, together with NSP, CP may play a key role in the pathogenicity of ToLCNDV in tomatoes. Moreover, the results revealed that different amino acids are involved in the pathogenicity of CPs. Sequence alignment showed that ToLCNDV-In and ToLCNDV-ES CPs shared 96.8% similarity, differing by only eight amino acids. Analysis of a series of point mutations revealed that alteration of arginine at the 143^rd^ amino acid position in the CP was sufficient for infectivity of ToLCNDV-ES. Point mutations affecting the 2^nd^, 29^th^, 32^nd^, 42^nd^, 148^th^, 193^rd^ or 194^th^ amino acid residue had no effect on infectivity of ToLCNDV-ES, which was never detected by PCR. ToLCNDV-ES became infectious in tomato only when tryptophan (W) was substituted by arginine (R) at position 143 in the CP. Sequence alignment revealed that other ToLCNDVs, which can infect tomatoes, also contained arginine at the 143^rd^ position while other amino acid residues were the same as those of ToLCNDV-ES in this study, indicating that this residue was critical for Mediterranean ToLCNDV (data not show). However, a single mutation of amino acid residue at 143-position of ToLCNDV-In CP still induced leaf curling and mosaic symptom, suggesting that the 143^rd^ amino acid residue alone is insufficient to determine the infectivity of ToLCNDV-In. Taken together, the data showed that when viruses of different genotypes were inoculated into the same host, their competence for infection varied, and these genetic variations may be the result of adaptations to new selection pressures during the transmission of ToLCNDV to a new area.

Proving to be a key determinant of ToLCNDV infectivity in tomatoes, the CP of ToLCNDV-ES could be disrupted primarily in the replication process and affect virus viability. The possibility of a virus infecting and inducing symptoms in a specific host plant depends on the expression of specific sets of genes and interactions between the host and virus-encoded proteins (Maule et al., 2002). Thus, yeast two-hybrid screening was performed to identify host proteins that interact with CPs to study the molecular mechanisms involved in the pathogenicity of the two ToLCNDV isolates. Five host proteins were captured as ToLCNDV-In CP bait-interacting partners, while the ToLCNDV-ES bait interacted with the other five host proteins. The binding test using yeast two-hybrid assays showed that all proteins interacted with ToLCNDV-In and ToLCNDV-ES CP. Interestingly, Ring finger protein 44-like, which was identified as an ES CP partner, did not bind to the India CP. Ring finger proteins are widely involved in the regulation of various physiological and biochemical processes, including plant growth and development, stress resistance, and hormone signaling responses (Borden, 2000; Sun et al., 2019). Ring finger proteins, a large family of E3 type, exist widely in eukaryotes and play important roles in ubiquitination, a central process of the ubiquitin/26S proteasome system that plays a role in plant–pathogen interactions (Zeng et al., 2006; Citovsky et al., 2009; Trujillo and Shirasu, 2010). This post-translational modification can affect geminiviral infections. For example, the Tobacco RING E3 Ligase NtRFP1 was reported to mediate the ubiquitination and degradation via the ubiquitin/26S proteasome system of the tomato yellow leaf curl China virus βC1 gene. After viral infection, plants overexpressing NtRFP1 developed attenuated symptoms, whereas plants with silenced expression of NtRFP1 showed severe symptoms (Shen et al., 2016). Therefore, our hypothesis is that the interaction of Ring finger protein 44- like protein with ToLCNDV-ES CP may disrupt the infection of tomatoes by this isolate. Expression of Ring finger protein 44- like in the two ToLCNDV-inoculated leaves did differ, and their upstream and downstream genes showed significantly changed expression levels under the two ToLCNDV-inoculated conditions. In addition to the potential degradation process of the Ring finger protein, the interaction of ToLCNDV-ES CP and Ring finger 44-like protein influences the gene in the upstream region and increases the transcript level, leading to a greater accumulation of defense compounds, suggesting a potential reason for ToLCNDV-ES infectivity in tomato. However, further studies are still required to verify that CP might be a substrate for Ring finger protein, as well as the impact of this protein on ToLCNDV infection in tomatoes.

Our study provides novel insights in understanding the impact of CPs on the difference in the pathogenicity of two ToLCNDV isolates, Asian and Mediterranean strains, in tomatoes. In addition, comparison of amino acids essential for host cell infectivity of ToLCNDV-ES and ToLCNDV-In provides clues of evolutionary conservation. Moreover, based on these data, various strategies can be applied in practice to benefit agriculture, especially for plant protection from plant viruses. Many studies using CPs to produce transgenic plants that show resistance to viruses were reported in the early 1990s. For example, tomato plants transformed with the TYLCV CP were found to be virus resistant (Kunik et al., 1994; Raj et al., 2005). Although further studies on biological and molecular properties are required, these findings have many advantages in the “breeding for resistance” research to prevent pathogen invasion in different geographical regions.

## Data availability statement

The original contributions presented in the study are included in the article/**Supplementary Material**, further inquiries can be directed to the corresponding author/s.

## Author contributions

GP, E-JK and SL conceived and developed the concept, supervised the experiments. TV, AL, BN, MT, MQ and ET performed the experiments. TV, GP, E-JK and SL contributed to the data analysis, interpretation, discussion and write the manuscript. All authors contributed to the article and approved the submitted version.
