# Supplementary material for: Coat protein is responsible for tomato leaf curl New Delhi virus pathogenicity in tomato
Source: Front Plant Sci. 2023 Jul 10;14:1206255. doi: 10.3389/fpls.2023.1206255 (PMC10364049; doi:10.3389/fpls.2023.1206255)

Supplementary Material

Coatprotein is responsible for tomato leaf curl New Delhi virus pathogenicity in tomato

Thuy T.B Vo^1,2^, Aamir Lal^3^, Bupi Nattanong^1^, Marjia Tabassum^1^, Muhammad Amir Qureshi^1^, Elisa Troiano^4^, Giuseppe Parrella^4,*^, Eui-Joon Kil^3,5,*^, Sukchan Lee^1, *^

*** Correspondence:**Dr.Giuseppe Parrella
giuseppe.parrella@ipsp.cnr.it

Prof. Eui-Joon Kil
viruskil@anu.ac.kr

Prof. Sukchan Lee

cell4u@skku.edu

# Supplementary Tables

**
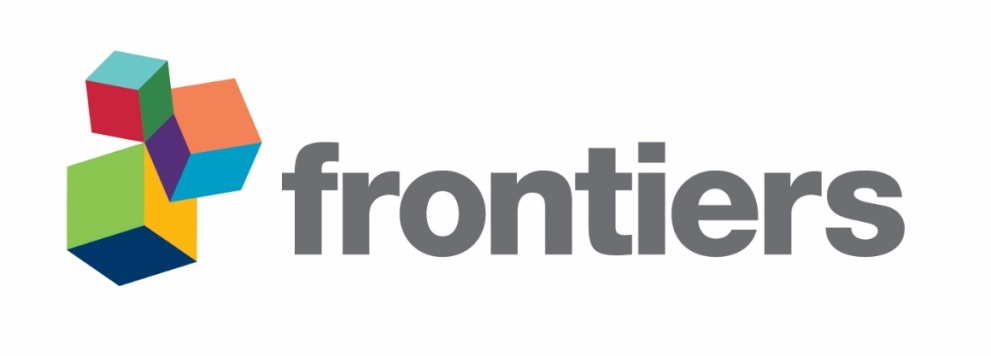
**

Table S1: Primer set used for site-directed mutant clones construction

| **Purpose** | **Primer** | **Sequence (5’-3’)** |
| --- | --- | --- |
| Mutation on  ToLCNDV-ES coat protein | IV1-2-F | AAGAACTATGGCGAAGCGACCAG |
|  | IV1-2-R | GGCTCGGAGAACTAAAATGAG |
|  | IV1-29-F | CCCATATGGAGCCCGTGCCTCTG |
|  | IV1-29-R | CTGTCGAAGTTGAGCCGTCG |
|  | IV1-32-F | AACCCGTGCCGTTGTCCCCATTG |
|  | IV1-32-R | CCATATGGGCTGTCGAAG |
|  | IV1-42-F | CACAAAAGCAAAGGCCTGGACGAAC |
|  | IV1-42-R | ACGCGGGCAATGGGGACA |
|  | IV1-143-F | TGTTCGTGACCGGCGTCCAACAG |
|  | IV1-143-R | AGAAAGAACATAACACTATTAGTATGATTTTTCG |
|  | IV1-148-F | TCCAACAGGATCCCCTCAGGAT |
|  | IV1-148-R | CGCCAGTCACGAACAAGA |
|  | IV1-193-F | AACGTATGCATGCAGGGAGCAAGC |
|  | IV1-193-R | CCTCCCGTCACAGTAGCA |
|  | IV1-194-F | GTATGCATCAAAGGAGCAAGCAT |
|  | IV1-194-R | GTTCCTCCCGTCACAGTA |
| Mutation on ToLCNDV-India coat protein | PaV1-2-F | AAGAACTATGTCGAAGCGACCAG |
|  | PaV1-2-R | TGCTCGGAGGACAAGAAT |
|  | PaV1-29-F | CCCCTATGGAACCCGTGCAGTTG |
|  | PaV1-29-R | CTGTCGAAGTTGAGACGTC |
|  | PaV1-32-F | AGCTCGTGCATCTGTCCCCATTGC |
|  | PaV1-32-R | CCATAGGGGCTGTCGAAG |
|  | PaV1-42-F | CACAAAAGCAAGGGCCTGGACAAACAG |
|  | PaV1-42-R | ACGCGGGCAATGGGGACA |
|  | PaV1-143-F | TTTGGTCCGTGACTGGCGTCCTACTGGATC |
|  | PaV1-143-R | GATCCAGTAGGACGCCAGTCACGGACCAAA |
|  | PaV1-148-F | TCCTACTGGAACCCCCCAAGATTTT |
|  | PaV1-148-R | CGACGGTCACGGACCAAA |
|  | PaV1-193-F | AACTTATGCAAGTAAGGAGCAAGCATTAG |
|  | PaV1-193-R | CCTCCCGTCACAGTCGCA |
|  | PaV1-194-F | TTATGCATGTAGGGAGCAAGCATTAG |
|  | PaV1-194-R | GTTCCTCCCGTCACAGTC |

Table S2: Primer sets used for expression of up and down stream genes of Ring finger protein 44-like by qRT-PCR

| **Primer** | **Sequence (5’-3’)** |
| --- | --- |
| Up-1-qRT-F | AAGCAAAGCGTGGAGAATGT |
| Up-1-qRT-R | AAATGCTGACAGGGATCCAG |
| Up-2-qRT-F | AAGACAAATGGGCCAAACAC |
| Up-2-qRT-R | AGGTCTGGCCATACATCGAG |
| Up-3-qRT-F | TGCGATGTGGTTTAGGTTCA |
| Up-3-qRT-R | GTGGTACGTGGGCTAGGAAA |
| Down-1-qRT-F | CTGGACTCAGCCAGGAACTC |
| Down-1-qRT-R | CGCCTTGATAGCAGGAAAAG |
| Down-2-qRT-F | TTATTATCGGGGAGCTGTCG |
| Down-2-qRT-R | CAGTTGAAACAGCCACGAGA |

Table S3: Infectivity of point mutant constructs on tomato

| ToLCNDV-ES CP | | | ToLCNDV-India CP | | |
| --- | --- | --- | --- | --- | --- |
| Mutant | Infectivity | Symptom | Mutant | Infectivity | Symptom |
| ESV1^S2R^ | 0/7 | -- | InV1^R2S^ | 7/7 | Leaf curling, yellow mosaic |
| ESV1^T29A^ | 0/7 | -- | InV1^A29T^ | 7/7 | Leaf curling, yellow mosaic |
| ESV1^S32V^ | 0/7 | -- | InV1^V32S^ | 7/7 | Leaf curling, yellow mosaic |
| ESV1^R42K^ | 0/7 | -- | InV1^K42R^ | 7/7 | Leaf curling, yellow mosaic |
| ESV1^W143R^ | 6/7 | No symptom | InV1^R143W^ | 7/7 | Leaf curling, yellow mosaic |
| ESV1^T148S^ | 0/7 | -- | InV1^S148T^ | 7/7 | Leaf curling, yellow mosaic |
| ESV1^S193C^ | 0/7 | -- | InV1^C193S^ | 7/7 | Leaf curling, yellow mosaic |
| ESV1^R194K^ | 0/7 | -- | InV1^K194R^ | 7/7 | Leaf curling, yellow mosaic |

Table S4: : Up and down stream genes of Ring finger protein 44-like

| **Position** | **Gene** | **NCBI number** | **Function** |
| --- | --- | --- | --- |
| Up stream | Mpv17 | XM_004243216 | Arabidopsis homolog enhances osmotic stress tolerance in plant **(Wi et al, 2020)** |
|  | Cytochrome P450 CYP72A219-like | XM_026032230 | Involved in growth, development and defense mechanism also induces biosynthesis of BR, JA and flavonoids **(Vasav and Barvkar et al, 2019)** |
|  | TMV resistance protein N-like | XM_004243735 | Regconize and trigger signal transduction leading to the defence response (**Marathe R et al, 2002**) |
| Down  stream | ATP-dependent zinc  metalloprotease FTSH 2 | XM_004243218 | Involved in the degradation and assembly of several thylakoid protein, also plays a role in protein quality control during nutrient deficiency **(Yesuke Kato et al, 2018)** |
|  | Rab11 GTPase | NM_001246869 | Important components of membrane trafficking also in protecting plants from damage induced by biotic and abiotic stress condition **(Manas K.T et al, 2021)** |

**Supplementary figures**

**Figure S1**: Diagram of chimeric DNA A component construction between two ToLCNDV (A): Scheme of infectious clones’ construction with tandem repeat construct. (B) Chimeric constructs between two ToLCNDV DNA A segments. Each ORF and IR of ToLCNDV-India was cloned into ToLCNDV-ES backbone and chimeric clones were generated


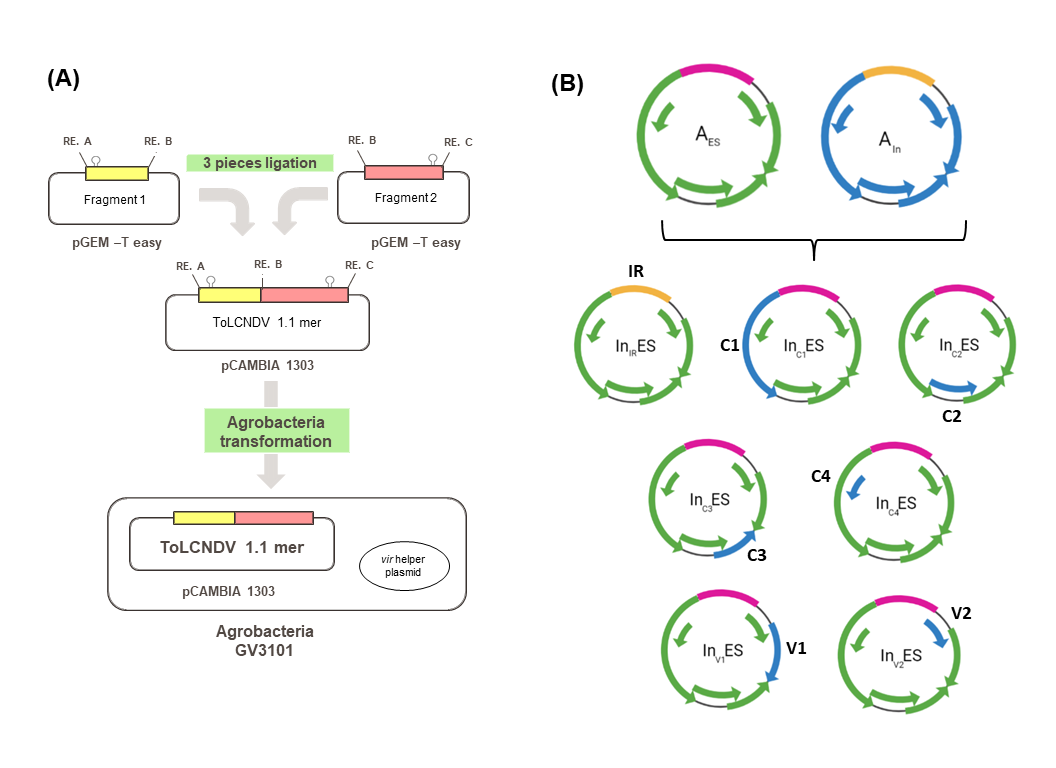


**Figure S2**: Position of different amino acids between two ToLCNDV coat protein. ToLCNDV-India coat protein (In CP) and ToLCNDV-ES coat protein (ES CP) sequence showed eight different amino acids at position 2,29,32,42,143,148, 193 and 194. The 16-point mutant clone was constructed based on these amino acids using the commercial Q5 kit.


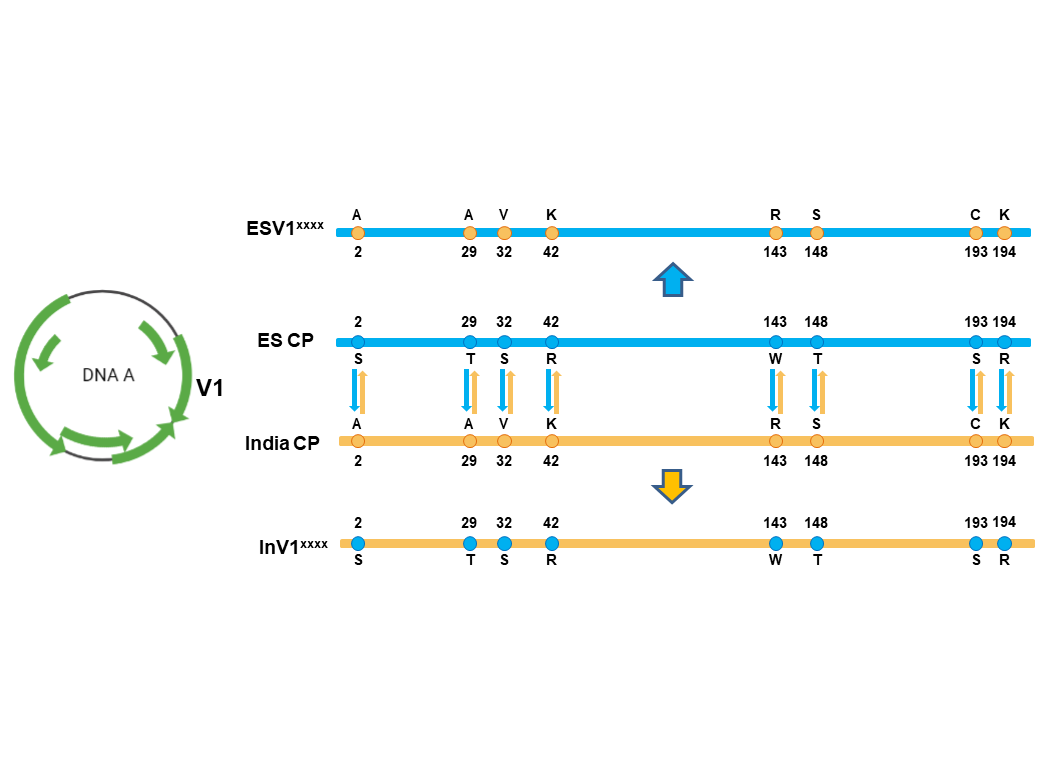

Supplement: Supplementary file 1 [file DataSheet_1.docx]
